# Evaluation of functional plant growth-promoting activities of culturable rhizobacteria associated to tunicate maize (*Zea mays* var. *tunicata* A. St. Hil), a Mexican exotic landrace grown in traditional agroecosystems

**DOI:** 10.3389/fmicb.2024.1478807

**Published:** 2024-10-02

**Authors:** Geovanny Rivera-Hernández, Guillermo Daniel Tijerina-Castro, Sandra Cortés-Pérez, Ronald Ferrera-Cerrato, Alejandro Alarcón

**Affiliations:** Microbiología de Suelos, Posgrado de Edafología, Colegio de Postgraduados, Montecillo, Mexico

**Keywords:** plant growth-promotion, biocontrol, functional microbial diversity, autochthonous microorganisms, native maize race

## Abstract

Tunicate maize (*Zea mays* var. *tunicata* A. St. Hil) is a landrace that constitutes a fundamental aspect of the socio-cultural identity of Ixtenco, Tlaxcala (Mexico) and represents an exotic phenotype whose kernels are enclosed in leaflike glumes. Despite multiple studies conducted worldwide on plant growth-promoting-rhizobacteria (PGPR) in commercial maize varieties grown under monoculture systems, very little is known about bacteria inhabiting native maize landraces in agroecosystems, but for tunicate maize such knowledge is non-existent. This research described and profiled functional groups of culturable rhizobacteria from tunicate maize at two phenological stages (tasseling and maturity/senescence) in a polyculture system, highlighting potential PGPR for biotechnological purposes. Ninety-five rhizobacteria were isolated and molecularly identified, and their physiological activities such as plant growth promotion, production of exogenous lytic enzymes, and antagonism against fungal pathogens were determined. The culturable rhizobacterial community associated to tunicate maize comprised 42 genera, dominated by Bacillaceae, Comamonadaceae, Microbacteriaceae, Micrococcaceae, Oxalobacteraceae, Pseudomonadaceae, and Rhizobaceae families. At tasseling stage, the identified bacteria corresponded to *Arthrobacter*, *Priestia*, *Herbaspirillum*, *Pseudomonas*, and *Rhizobium*, and exhibited redundant capabilities for stimulating plant growth and nutrition, and inhibiting fungal phytopathogens. At maturity/senescence stage, the main genera *Arthrobacter* and *Microbacterium* displayed lytic capabilities to support mineralization process. We recorded potential novel rhizosphere functional bacteria such as *Rhizobium*, *Sphingobium*, and *Arthrobacter* which are not previously described associated to maize landraces, as well as their bioprospection as PGPR detected at plant phenological stages poorly explored (like maturity/senescence). This taxonomic and functional diversity was attributed to the application of agricultural practices as well as the rhizosphere effect during specific phenological stages. Results described the diversity and functionality of culturable rhizosphere bacteria from tunicate maize in polyculture systems that allowed us the detection of potential rhizobacteria for further developing of biofertilizers and biocontrollers directed as biotechnology for sustainable agriculture, and for generating strategies for conservation of native plants and their microbial genetic resources.

## Introduction

Mexico is considered the center of origin and domestication of maize (*Zea mays* spp. *mays*). Fifty-nine native maize races and numerous landraces with high genetic diversity, perfectly adapted to various climates and soil conditions, have been documented (CONABIO, 2011; Perales and Golicher, 2014). Tunicate maize is a landrace exclusively cultivated in traditional agroecosystems in San Juan Ixtenco, located in the central Mexican highlands in Tlaxcala, Mexico, and holds significant cultural importance for the local community (Maria-Ramirez et al., 2017). It is characterized by an exotic phenotype in which each kernel, round-shaped with an extended pedicel, is completely enveloped by leaf-like glumes. Additionally, the male inflorescence of plants (tassel) is elongated and feminized, displaying the presence of grains. This maize is considered an endangered variety, and its current presence is the result of the resistance and conservation commitment of a few local farmers (Carranza et al., 2016; Sangermán-Jarquín et al., 2018). However, in recent years, there has been a prevailing trend of large-scale cultivation of hybrid maize varieties, which are chemically fertilized, leading to a significant reduction of native maize cultivars (Mclean-Rodríguez et al., 2019; Guzzon et al., 2021). If this trend continues, the biotechnological potential of autochthonous microorganisms associated to native maize varieties is also at risk of disappearing, without being able to be harnessed for sustainability objectives.

On the other hand, given the environmental challenges associated with current intensive food production systems (Wang et al., 2018), the application of plant growth-promoting rhizobacteria (PGPR) has emerged as a viable alternative, due to their ability to exert beneficial effects on plants and enhance agronomic yields in a sustainable manner (Upadhyay et al., 2022). These bacteria possess various direct and indirect mechanisms for promoting plant growth (Backer et al., 2018). Direct mechanisms include the solubilization of phosphorus and potassium, mobilization of organic phosphorus, siderophore production, and biological nitrogen fixation (BNF), which enhance the acquisition and mobilization of nutrients vital to the plant. Additionally, they can stimulate growth and modify plant architecture by producing phytohormones such as auxins, gibberellins, and cytokinins. Conversely, indirect mechanisms involve conferring resistance to phytopathogens via antagonistic capabilities, either through the production of exogenous lytic enzymes (such as chitinases, cellulases, and proteases), the synthesis of secondary metabolites (such as antibiotics, antifungal and hydrogen cyanide), or competition for essential soil nutrients (siderophores). Furthermore, PGPR improves tolerance to abiotic stresses by producing ACC deaminase, and synthesizing osmolytes and exopolysaccharides (Di-Benedetto et al., 2017; Backer et al., 2018; Gouda et al., 2018; Upadhyay et al., 2022).

Bacterial genera like *Achromobacter*, *Arthrobacter*, *Azospirillum*, *Bacillus, Brevundimonas*, *Bradyrhizobium*, *Burkholderia*, *Klebsiella*, *Herbaspirillum*, *Pantoea, Pseudomonas*, and *Rhizobium* are frequently described as PGPR to maize, especially on hybrid commercial varieties (Roesch et al., 2007; Arruda et al., 2013; Kifle and Laing, 2016; Alves et al., 2020; Ercole et al., 2021). In contrast, the understanding of the structure and function of soil bacterial biodiversity in traditional Mesoamerican agroecosystems, known as *milpa*, and the potential of PGPR isolated from native maize landraces cultivated in these environments has been relatively underexplored (Aguirre-von-Wobeser et al., 2018). Among the few studies that exist in Mexican agroecosystems, one focused on the recruiting of a diazotrophic community of a maize landrace from the Sierra Mixe, Oaxaca (Higdon et al., 2020), while another examined the rhizospheric and endophytic bacteria with growth-promoting potential in the giant native maize from Jala, Nayarit (Rios et al., 2021). Interestingly, despite the limited studies on PGPR cohabiting within native maize landraces, research based on metagenomic approaches suggests that the genomic variations inherent to these maize varieties are associated with differences in the diversity of bacterial communities (Lund et al., 2022). Furthermore, the composition and function of the rhizobiome in these native varieties exhibit better adaptive capacities to agroecosystems compared to modern maize (Schmidt et al., 2020). Likewise, *milpas* may preserve ancient plant-microorganism interactions that could have been lost in modern monocultures characterized by high tillage and large agrochemicals inputs (Aguirre-von-Wobeser et al., 2018). Additionally, the maize plant, throughout its various growth stages, continually influences the assembly and functionality of the microbiome, stimulating functional microbial groups capable of exerting physiological and ecological roles (Xiong et al., 2021).

Understanding the functional diversity of rhizobacteria associated with maize varieties growth in Ixtenco-*milpa* under a culturable-dependent approach could facilitate the development of customized bioformulations tailored to local agroecosystems, particularly for application in the sustainable intensification of production systems in the central Mexican highlands. We hypothesize that tunicate maize harbors a taxonomically and functionally diverse community of culturable indigenous rhizobacteria, shaped by edaphic and phenological conditions, with *in vitro* PGPR activities involved to the nutrition, health, and ecology of the plant. Thus, represents an unexplored source of viable microorganisms and an initial step toward their formulation as biofertilizers and biocontrollers for this cereal. In this study, we explored and described the culturable rhizobacterial populations identified during the tasseling and physiological maturity/senescence stages of tunicate maize, emphasizing their possible roles as PGPR, such as producing auxins (IAA, indole-3-acetic acid) or siderophores, solubilizing phosphate (PS), growing in nitrogen-free media (NFb), releasing lytic enzymes, and inhibit fungal phytopathogens.

## Materials and methods

### Site description and sample collection

**Figure 1:**
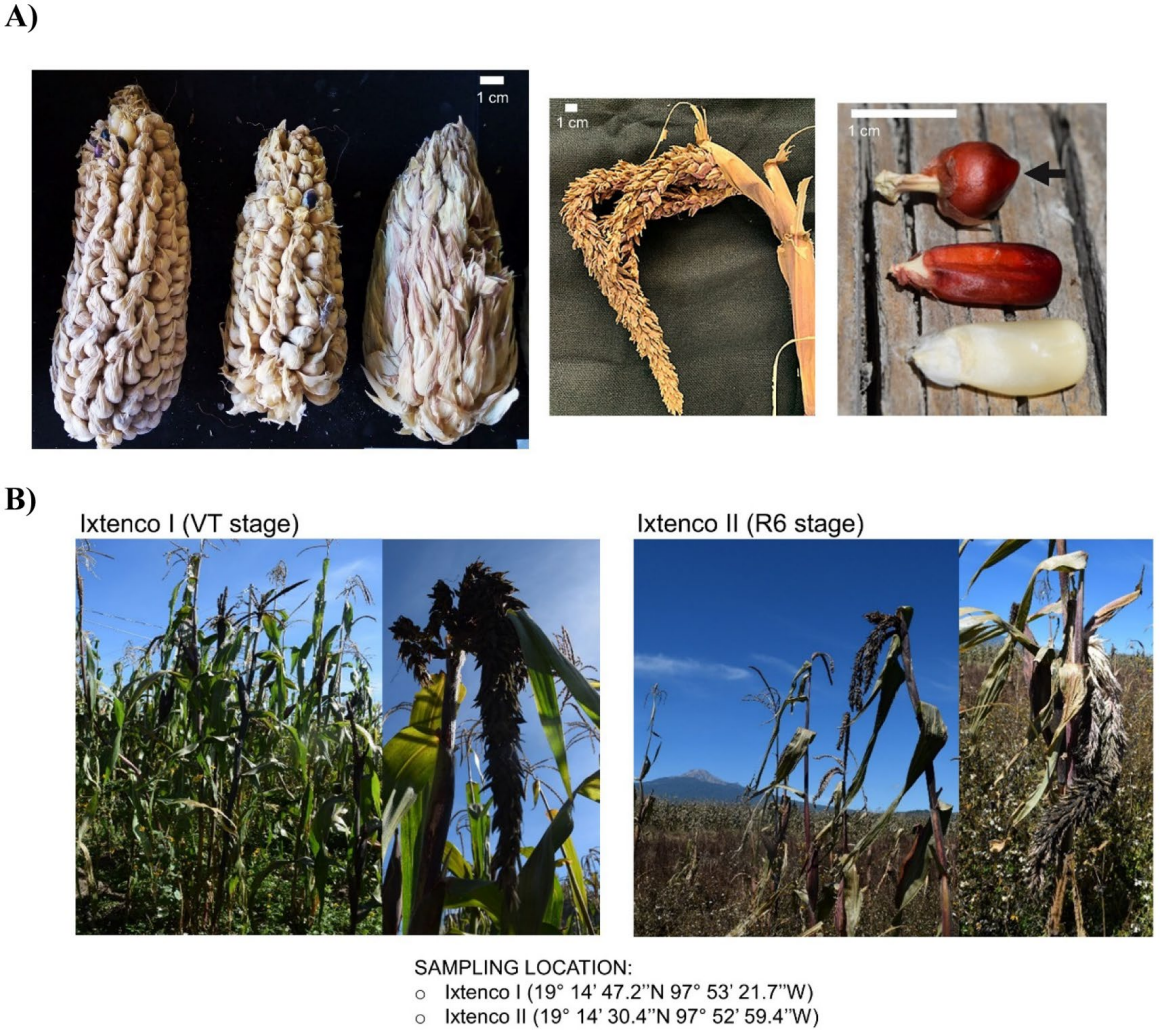
Phenotype of tunicate maize plant and sampled sites. **(A)** Phenotypic features of cob, tassel, and kernel (indicated by black arrow) from tunicate maize. **(B)** Location and phenological stages of the sampled plants. Plants from Ixtenco I were in the phenological tasseling stage (VT), while those from Ixtenco II were in the maturity/senescence stage (R6).

Rhizosphere soil samples were collected on October 4, 2020, from two locations in San Juan Ixtenco, situated in the central Mexican highlands of the state of Tlaxcala (~2,500 masl), characterized by a temperate subhumid climate with summer rainfall. Maize production in Ixtenco region, is rainfed and agronomical practices involve the use of agroecosystems, called *milpa,* organically fertilized through the incorporation of crop residues and livestock manure into the soil, crop rotation, and reduced application of chemical inputs and pesticides. Similar farming practices are employed at both sampled locations. Sampling was conducted with the collaboration and permission of local farmers. Tunicate maize plants were identified based on the distinctive phenology of the tassel and cob formation (Figure 1A). A total of six rhizospheric soil samples were collected, with three samples per plot. Both locations are near each other (~840 m apart) and belong to the same edaphoclimatic zone.

Location Ixtenco I (19°14′ 47.2″ N, 97°53′ 21.7″ W) is a small *milpa* (20 × 40 m) situated on the outskirts of the urban area. The tunicate maize plants were identified in the tasseling (VT) phenological stage within a polyculture system in where Curcubitaceae (*Cucurbita ficifolia* and *Cucurbita pepo*), Ayocote beans (*Phaseolus coccineus*), Acahual (*Simsia amplexicaulis*), Mozoquelite (*Bidens pilosa*), and native maize varieties coexist.

Location Ixtenco II (19°14′ 30.4″ N, 97°52′ 59.4″ W) is a larger *milpa* (60 × 200 m) located outside the urban area. This plot is also a polyculture system, primarily with Curcubitaceae and Ayocote beans, where the plants were found in the maturity/senescence stage (R6) (Figure 1B).

Rhizosphere soil samples were taken at 20 cm-depth around the root, manually shaken for 2 min to remove loose soil, and the remaining attached-soil to the roots was collected in polyethylene bags, and subsequently stored at 4°C for 2 days until processing them.

### Physical and chemical analysis of soil samples

Soil samples were air-dried, homogenized, and sieved through a 2 mm mesh. The assessed physical and chemical properties included texture (hydrometer method) (Bouyoucos, 1962), pH (aqueous solution method), electrical conductivity (EC) (saturation extract method) (Kalra and Maynard, 1991), soil organic matter (SOM) (Walkley and Black, 1934), total nitrogen (TN) (micro-Kjeldahl method) (Bremner, 1996), available phosphorus (P) (Bray and Kurtz, 1945), cation exchange capacity (CEC) and exchangeable potassium (K) (ammonium acetate method) (FAO, 2022). These properties were determined at the Soil Genesis, Morphology and Classification Laboratory Edaphology Department (Colegio de Postgraduados, Mexico).

### Isolation of culturable rhizobacteria

Serial dilutions (1 × 10^−1^ to 1 × 10^−5^) of rhizospheric soil were prepared in sterile distilled water, and 100 μL aliquots of each dilution were plated onto nutrient agar (Merck^(R)^) and yeast mannitol agar (YMA) with red Congo dye (Vincent, 1970). Plates were incubated for 2–5 days at 28°C. Based on the bacterial growth, colonies from each phenological maize stage were isolated according to their colonial morphology. Colonies with similar morphology were considered the same bacteria, while distinct colony morphologies indicated different microorganisms. Purity of bacterial isolates was confirmed using the quadrant streaking method (at least 5 times) on both nutrient agar and YMA plates, followed by microscopic examination. Subsequently, bacterial isolates were preserved in 20% glycerol and slant tubes of nutrient agar, and stored at 4°C.

### Amplification of the 16S rRNA gene, sequencing, and molecular identification

Bacteria isolated at each phenological stage, displaying distinct colonial morphologies, were molecularly identified by sequencing the 16S rRNA gene.

Total bacterial genomic DNA was extracted by CTAB method (Doyle et al., 1990). The 260/280 nm ratio evaluated the concentration and quality of the extracted DNA. The 16S rRNA gene was amplified by PCR using universal primers 16S rRNA: 27F (5′-AGAGTTTGATCMTGGCTCAG-3′) and 1492R (5′-GGTTA CCTTGTTACGACTT-3′), under the following conditions: denaturation at 96°C for 1 min, followed by 35 cycles at 96°C for 30 s, 50°C for 1 min, 60°C for 1.5 min, and a final extension at 60°C for 4 min. The amplified product obtained (~1,400 bp) was verified on a 1% agarose gel and purified with EXO-SAP (Affymetrix, United States) following the instructions provided by the manufacturer. The fragment was sequenced using the services of Psomagen Inc. (United States).

The generated sequences were aligned and corrected using Geneious software to obtain consensus sequences, which were then compared to the National Center for Biotechnology Information database using BLAST (Basic Local Alignment Search Tool) (Altschul et al., 1990). The phylogenetic analysis was carried out by comparing each consensus sequence with its respective reference sequences compiled from the database available at http://www.ncbi.nlm.nih.gov/Genbank. The 16S rRNA gene sequences were aligned using the Muscle multiple alignment program incorporated into MEGA X software (Kumar et al., 2018). The phylogenetic tree was constructed with MEGA X using the Neighbor-Joining (NJ) method (Saitou and Nei, 1987) with 10,000 replicates employing the bootstrap analysis to obtain confidence values (Felsenstein, 1985). Evolutionary distances were calculated using the Kimura 2-parameter method (Kimura, 1980).

### Nucleotide sequence accession numbers

The nucleotide sequences of 16S rRNA were deposited in GenBank. The accession numbers of the 16S rRNA nucleotide sequences of the 95 strains are PP111633 to PP111727 and are presented (Supplementary Table S1).

### Functional characterization of bacterial isolates

The assessment of the biotechnological potential of bacterial isolates was conducted through qualitative *in vitro* assays focused on detecting PGP traits, production of exogenous lytic enzymes of biotechnological interest, and antagonistic capabilities against *Fusarium oxysporum*. For each bacterial isolate, three replicates were included for each assay. In those tests in where a halo was detectued around the bacterial colony; thus, a clearance index (CI) was estimated analogously to the solubilization index described below. Therefore, the presence of the evaluated capacity was correlated with CI values greater than 1.0, and qualified it as outstanding if CI was higher than 1.4.

For assessing auxin production, a colorimetric assessment was performed in 96-well microplates to detect the presence of indoles in liquid culture using the Salkowski reagent (Sarwar and Kremer, 1995).

Regarding phosphate solubilization, the ability of bacterial isolates for solubilizing phosphates was determined using Pikovskaya agar medium, containing tricalcium phosphate as P-source (Nautiyal, 1999). The solubilizing capacity is described by the solubilization index (SI), defined as the ratio of the total halo diameter to the colony diameter (Kumar and Narula, 1999).

The ability of bacteria to grow in a nitrogen-free medium serves as an indicator, revealing their potential for conducting biological nitrogen fixation (BNF). A screening to identify potential diazotrophic bacteria was performed using nitrogen-free semi-solid medium (NFb). The presence of putative diazotrophic bacteria was detected by observing the color change from green to blue and the formation of a pellicle in the subsurface of the medium, as described by Baldani et al. (2014).

The production of siderophores was carried out using the Chrome Azurol S (CAS) universal agar plate assay. Positive siderophore production was identified by detecting a yellow halo surrounding the bacterial colonies (Schwyn and Neilands, 1987).

The protease activity was assessed on skim milk agar medium and visualized by the development of a clear halo around the colonies (Naik et al., 2008).

The lipase production was observed using a lipase medium containing Tween 80 as substrate and Rhodamine B as indicator dye. Positive detection was assessed in strains exhibiting the presence of a halo around the bacterial colony (Smibert and Krieg, 1994).

The cellulolytic capacity was visualized by inoculating each bacterial isolate on nutrient agar plates added with carboxymethylcellulose. The positive detection of cellulase was assessed by detecting the presence of a halo around the bacterial colony as described by Slama et al. (2019).

The chitinase activity was assessed on 1% (w/v) colloidal chitin agar plates (Subramanian et al., 2020). The chitin hydrolysis was visualized by the formation of a clear zone around the bacterial colonies according to Saima and Roohi (2013).

The ability of bacterial isolates to inhibit the growth of *Fusarium oxysporum* was assessed through *in vitro* assays (Slama et al., 2019) on PDA plates incubated at 28°C for 14 days with periodic monitoring. The strain of *F. oxysporum* f. sp. *cubense* race 1 (CNRF-MIC17191) was obtained from the mycology laboratory of the Centro Nacional de Referencia Fitosanitaria (CNRF), belonging to the Servicio Nacional de Sanidad, Inocuidad y Calidad Agroalimentaria (SENASICA, México) (Hernández-Melchor et al., 2023). The following formula was employed to calculate the percentage of fungal growth inhibition (Mulk et al., 2022):


%inhibition=[(C−T)∕T]×100

Where “C” represents the diameter of fungal growth in the negative control, and “T” is the diameter recorded from the fungal growth with bacterial confrontation. A minimum inhibition of 20% was considered to classify the bacterial isolate as a good candidate as biocontrol agent.

### Data analysis of the *in vitro* functional assays

Data collected from *in vitro* assays, encompassing IAA biosynthesis, phosphate solubilization, growth in nitrogen-free medium, siderophore, cellulase, protease, and lipase production, were analyzed collectively.

The assignment of relative scores for assays ranged numerically from zero to two. A score of zero was assigned for the absence of the evaluated capacity, a score of one if it exhibited good capacity, and a score of two if the capacity was outstanding. For the biocontrol agent screening, the inhibition percentages obtained at the sixth day of the established periodic monitoring were considered.

The phylogenetic tree constructed in MEGA X was saved in Newick format and subsequently loaded onto the iTOL v5 (Interactive Tree Of Life) web server (Letunic and Bork, 2021) for visualization and annotation of assigned values in circular dendrograms with heatmaps.

## Results

### Physical and chemical properties of rhizosphere soil

Soil samples had pH and EC values ranging from 5.4 to 6.5, and 0.18 to 0.60 dS m^−1^, respectively, and were classified as slightly acidic soils with low salinity. Additionally, the soil organic matter (SOM) content ranged from 1.42 to 2.57%. A low total nitrogen (TN) content was detected and ranged from 0.113 to 0.021%; exchangeable potassium (K) content was low with values below 0.3 cmol_(+)_kg^−1^ in most of the samples. Regarding available phosphorus (P), the Ixtenco I sample exhibited a medium to high level (30–59 mg P kg^−1^), while the Ixtenco II sample had a low level of this nutrient (11–25 mg P kg^−1^). The CEC was very low (≤5 cmol_(+)_kg^−1^); finally, the texture of both soil samples was sandy-loam (Table 1). Therefore, the soil from tunicate maize of Ixtenco samples, presents nutrient deficiencies.

**Table 1:** Physical and chemical properties of tunicate maize rhizosphere soils.

Location	Sample	pH	EC (dS m^−1^)	SOM (%)	TN (%)	P (mg Kg^−1^)	CEC (cmol_(+)_ Kg^−1^)	K (cmol_(+)_ Kg^−1^)	Soil texture
Ixtenco I	1	6.5	0.34	1.80	0.042	30.3	3.30	0.19	Sandy loam
	2	5.8	0.29	1.67	0.049	37.1	3.10	0.29	Sandy loam
	3	5.5	0.60	2.57	0.113	59.0	5.24	0.69	Sandy loam
Ixtenco II	4	5.6	0.18	1.67	0.021	11.0	0.39	0.27	Sandy loam
	5	5.4	0.28	1.54	0.064	24.8	1.55	0.42	Sandy loam
	6	5.5	0.18	1.42	0.035	12.2	0.97	0.27	Sandy loam

EC, electrical conductivity; SOM, soil organic matter; TN, total nitrogen; P, available phosphorus; CEC, cation exchange capacity; K, exchangeable potassium.

### Molecular identification of 16S rRNA of culturable rhizobacteria

**Figure 2:**
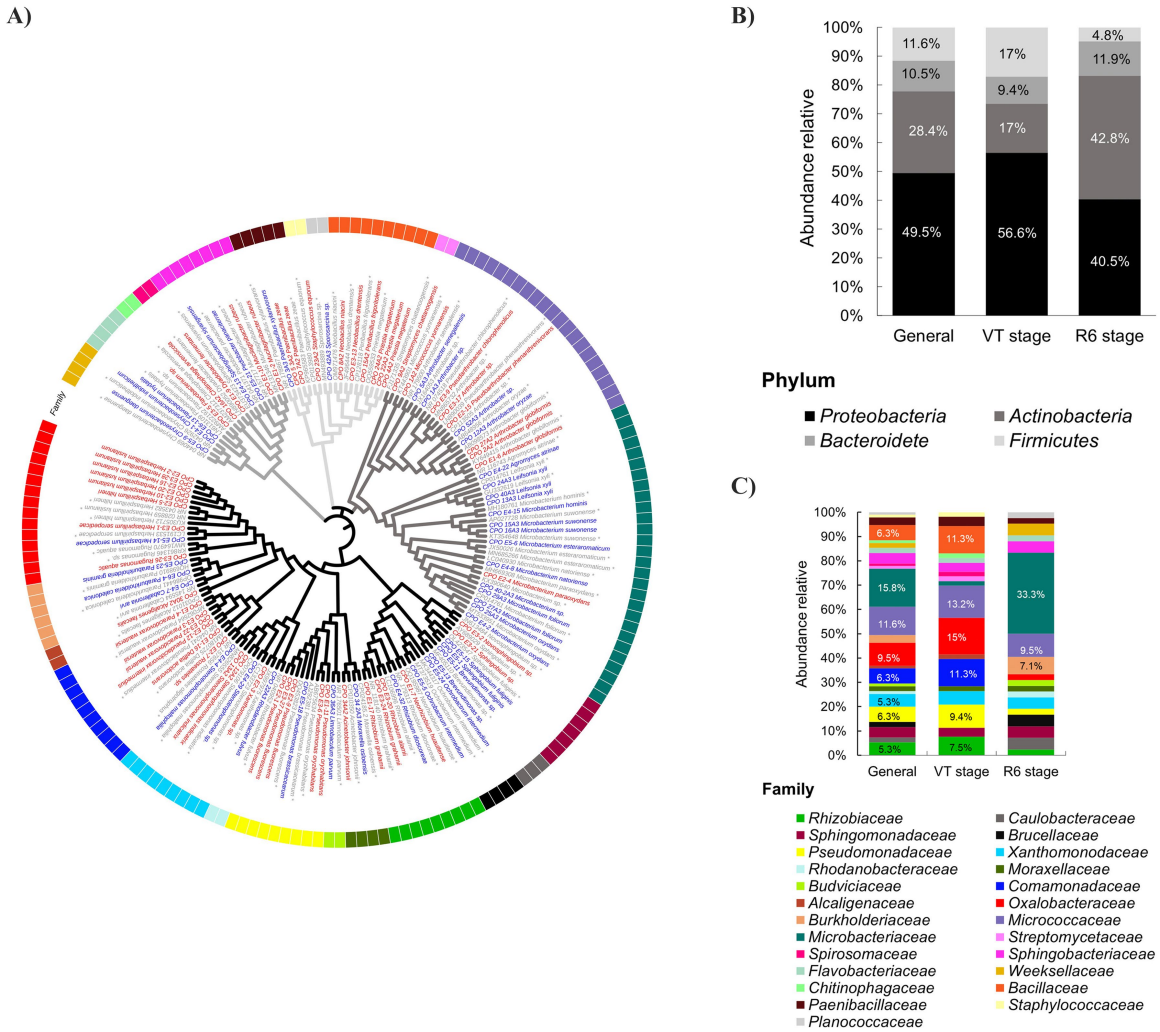
Phylogenetic tree based on the nucleotide sequence of 16S rRNA and relative abundance of rhizobacteria isolated from tunicate maize. **(A)** The phylogenetic tree was constructed using MEGA X software with the neighbor-joining (NJ) method and a bootstrap of 10,000 replicates. The consensus sequences of strains isolated from tunicate maize at the VT stage are marked in red, and those isolated at the R6 stage are marked in blue. Asterisks (*) indicate the reference sequences compiled from the GenBank database. Black, dark-gray, mild-gray, and light-gray branch colors correspond to Proteobacteria, Actinobacteria, Bacteroides, and Firmicutes phyla, respectively. Relative abundance of rhizobacterial isolated taxa at **(B)** phylum and **(C)** family levels. The bacterial phyla are depicted using distinct shades of gray-black in the upper graph of relative abundance. The bacterial families are delineated by different colors in the lower graph of relative abundance. General refers to the entire population of culturable rhizobacteria identified in this study (*n* = 95). VT stage (*n* = 53) and R6 stage (*n* = 42) are the phenological stages of the plant from which the rhizobacteria were isolated.

Initially, 186 rhizospheric bacteria were isolated. After being morphotypically differentiated, 95 of these bacteria were molecularly identified (Supplementary Table S1). The identified strains were classified into 42 genera belonging to 25 families across four main phyla: Proteobacteria, Actinobacteria, Firmicutes, and Bacteroidetes (Figure 2A).

The 25 families identified were Rhizobiaceae, Caulobacteraceae, Sphingomonadaceae, Brucellaceae, Pseudomonadaceae, Xanthomonodaceae, Rhodanobacteraceae, Moraxellaceae, Budviciaceae, Comamonadaceae, Alcaligenaceae, Oxalobacteraceae, Burkholderiaceae, Micrococcaceae, Microbacteriaceae, Streptomycetaceae, Spirosomaceae, Sphingobacteriaceae, Flavobacteriaceae, Weeksellaceae, Chitinophagaceae, Bacillaceae, Paenibacillaceae, Staphylococcaceae, and Planococcaceae (Figure 2C).

Among the *α*-Proteobacteria, the identified genera were *Rhizobium*, *Neorhizobium*, *Ochrobactrum*, *Brevundimonas*, *Novosphingobium*, and *Sphingobium*. As part of the *β*-Proteobacteria, the recorded genera corresponded to *Herbaspirillum*, *Rugamonas*, *Caballeronia*, *Paraburkholderia*, *Alcaligenes*, *Paracidovorax*, *Pseudacidovorax*, *Delftia*, and *Roseateles*. For the *γ*-Proteobacteria, the identified genera were *Stenotrophomonas*, *Xanthomonas*, *Rhodanobacter*, *Limnobaculum*, *Acinetobacter*, *Moraxella*, and *Pseudomonas*.

Within the phylum Actinobacteria the identified genera were *Streptomyces*, *Micrococcus*, *Arthrobacter*, *Pseudoarthrobacter*, *Agromyces*, *Leifsonia*, and *Microbacterium*. In the Firmicutes phylum, the identified genera were *Priestia*, *Peribacillus*, *Neobacillus*, *Sporosarcina*, *Staphylococcus*, and *Paenibacillus*. Finally, in the Bacteroidetes phylum, genera like *Chryseobacterium*, *Flavobacterium*, *Chitinophaga*, *Dyadobacter*, *Sphingobacterium*, *Pedobacter*, and *Mucilaginibacter* were identified.

### Taxonomic composition of the culturable rhizobacterial community

The culturable rhizobacterial community was predominantly composed of the phyla Proteobacteria, Actinobacteria, Firmicutes, and Bacteroidetes, which exhibited relative abundance of 49.5, 28.4, 11.6, and 10.5%, respectively (Figure 2B). Although the enrichment patterns of the dominant phyla (Proteobacteria: Actinobacteria) were consistent across isolates from both locations (Ixtenco I and Ixtenco II), notable differences were observed in their relative proportions. Specifically, the Proteobacteria:Actinobacteria ratio was 3:1 for Ixtenco I and 1:1 for Ixtenco II (Figure 2B).

The bacterial community isolated from Ixtenco I soil samples (*n* = 53), where plants were at the tasseling phenological stage (VT), was characterized by the prevalence of families Oxalobacteraceae (15%), Micrococcaceae (13.2%), Comamonadaceae (11.3%), Bacillaceae (11.3%), Pseudomonadaceae (9.4%), and Rhizobiaceae (7.5%). In contrast, in Ixtenco II (*n* = 42), where plants were at the maturity/senescence stage (R6), the family Microbacteriaceae (33.3%) was predominant, followed by families Micrococcaceae (9.5%) and Burkholderiaceae (7.1%) (Figure 2C). Therefore, the phenological state of the plant directs the assembly of the culturable rhizospheric community.

### Functional characterization of culturable rhizobacterial community

A total of 95 identified strains were qualitatively assessed for their PGP traits, exogenous lytic enzyme production, and antagonistic capabilities against phytopathogenic fungi.

**Figure 3:**
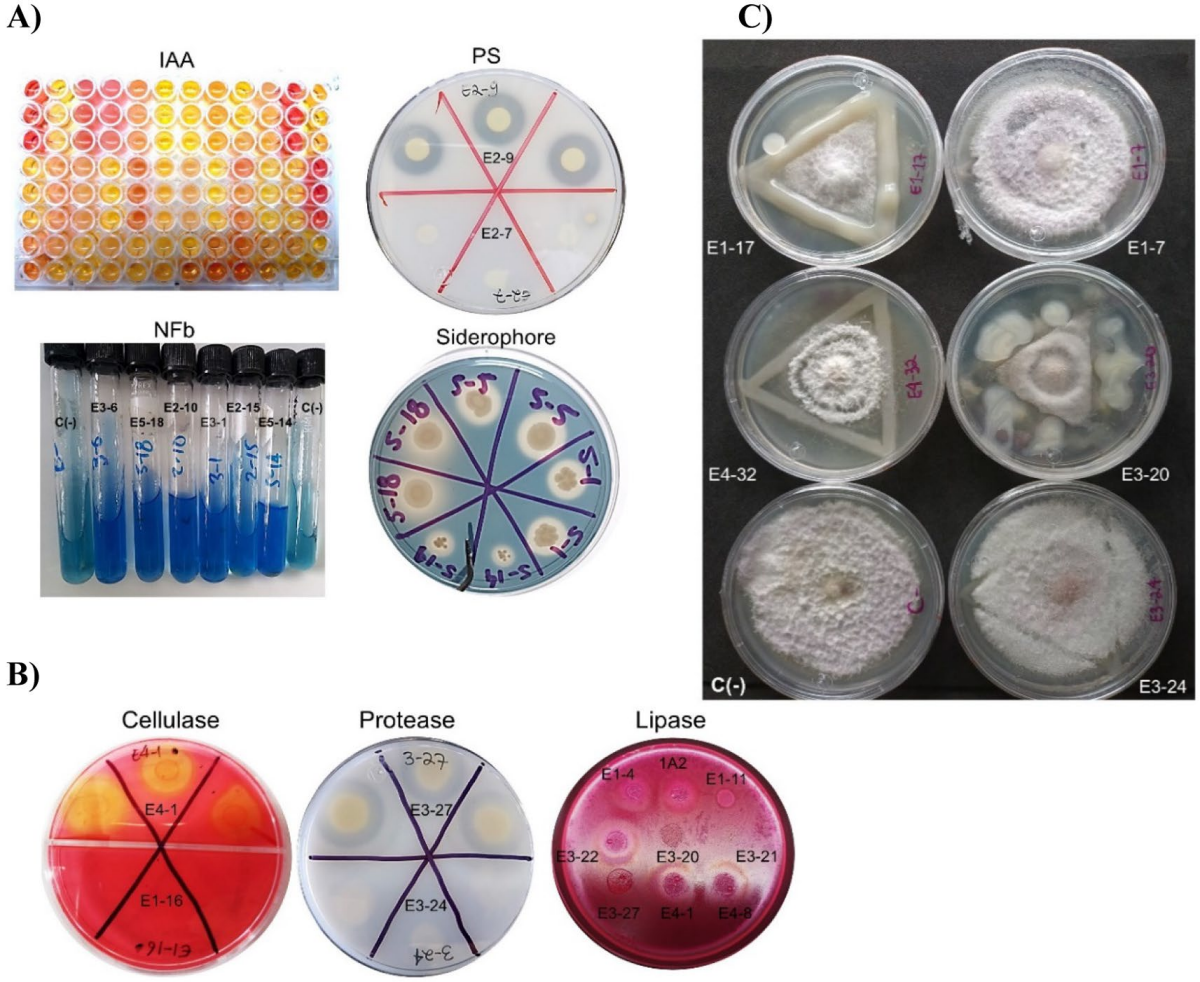
Detection of PGP traits, production of lytic enzymes, and antagonism against *Fusarium oxysporum* of rhizobacteria isolated from tunicate maize. **(A)** Qualitative *in vitro* assays focused on detecting PGP traits: biosynthetic capacity for auxin (IAA), phosphate solubilization (PS), ability to grow in nitrogen-free media (NFb), and siderophore production. C(−): negative control. **(B)** Production of exogenous lytic enzymes: cellulase, protease, and lipase. **(C)** *In vitro* antagonistic capabilities against *Fusarium oxysporum* f.sp. *cubense* race 1 (CNRF-MIC17191) at 6 days of confrontation, C(−): negative control. The negative control was *F. oxysporum* without bacteria. E1-4: *Paracidovorax wautersii*, E1-7: *Neorhizobium huautlense*, E1-11: *Pseudomonas oryzihabitans*, E1-16: *Delftia acidovorans*, E1-17: *Rhizobium grahamii*, 1A2: *Micrococcus yunnanensis*, E2-7: *Roseateles* sp., E2-9: *Pseudomonas fluorescens*, E2-10: *Herbaspirillum lusitanum*, E2-15: *Pseudarthrobacter phenanthrenivorans*, E3-1: *Herbaspirillum seropedicae*, E3-6: *Pseudomonas oryzihabitans*, E3-20: *Rhizobium alamii*, E3-21*: Sphingobium* sp., E3-22: *Paracidovorax wautersii*, E3-24: *Rhizobium grahamii*, E3-27: *Pseudomonas fluorescens*, E4-1: *Chryseobacterium indoltheticum*, E4-8: *Microbacterium natoriense*, E4-32: *Rhizobium dioscoreae*, E5-1: *Sphingobium fuliginis*, E5-5: *Ochrobactrum intermedium*, E5-14: *Herbaspirillum seropedicae*, and E5-18: *Pseudomonas brassicacearum*.

Regarding PGP traits, 54 strains (57%) were found to produce indoles, 25 strains (26%) exhibited visual activity as P-solubilizers, 18 strains (19%) were putative diazotrophs due to the ability to grow in nitrogen-free medium along with a color change to blue and pellicle formation in NFb medium, and 24 strains produced siderophores (25%) (Figure 3A).

In terms of lytic capabilities, 52 strains (55%) were cellulase producers, 62 strains (65%) produced protease, 21 strains (22%) were lipase producers, and no chitinase production was detected in any evaluated strain (Figure 3B).

**Figure 4:**
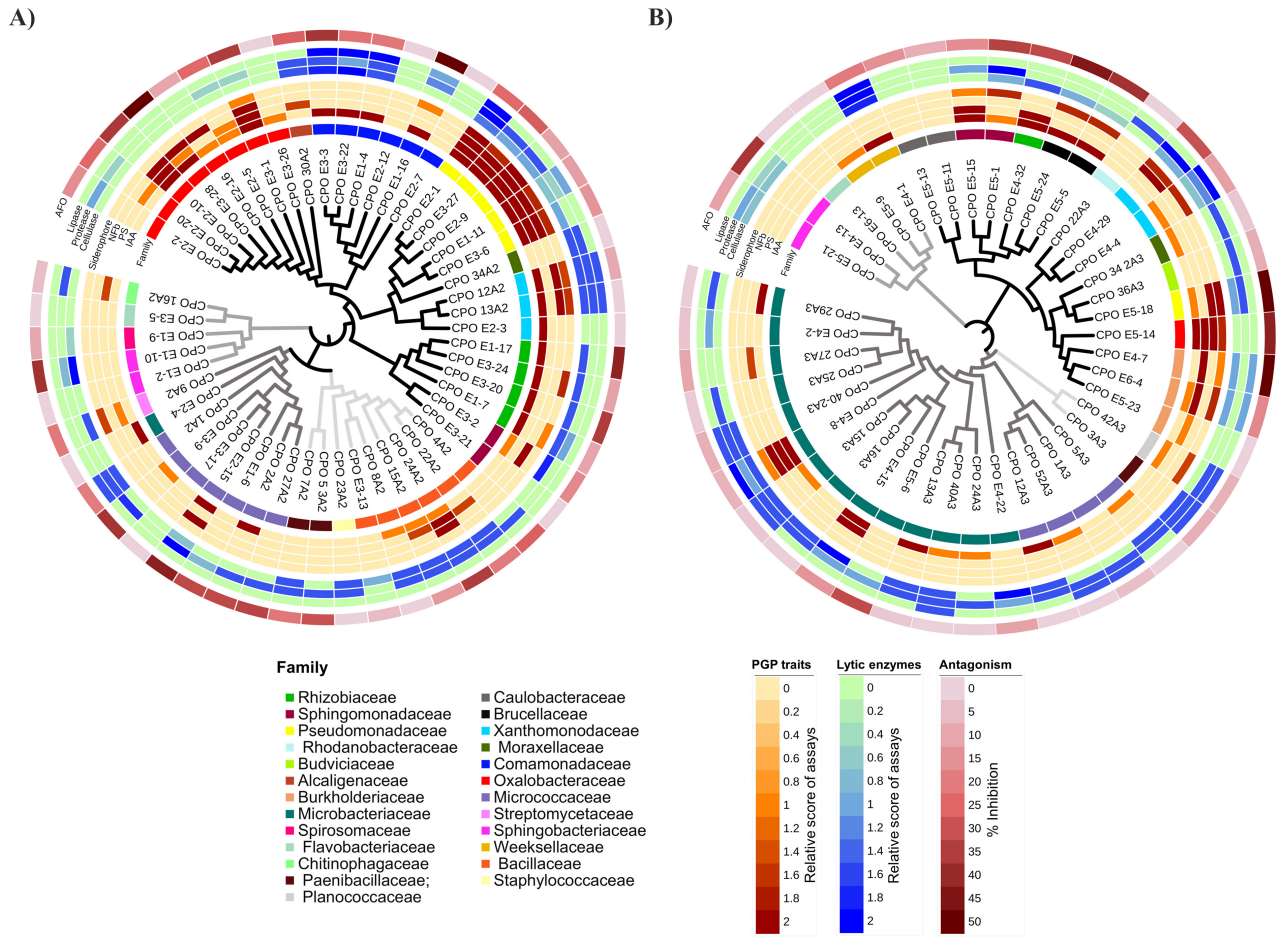
Dendrograms with heatmaps depicting PGP traits, lytic enzymes, and antagonism of rhizobacteria isolated from tunicate maize during the phenological stages of tasseling **(A)** and maturity/senescence **(B)**. Data from *in vitro* assays were presented as heatmaps alongside phylogenetic dendrograms, using phylogenetic trees constructed in MEGA X for each phenological stage and edited on the iTOL v5 (Interactive Tree of Life) web server. Black, dark-gray, mild-gray, and light-gray branch colors correspond to Proteobacteria, Actinobacteria, Bacteroidetes, and Firmicutes phyla, respectively. The first concentric ring around the dendrogram represents taxonomic families. The second ring depicts PGP traits: biosynthetic capacity for auxin (IAA), phosphate solubilization (PS), ability to grow in nitrogen-free media (NFb), and siderophore production. The third ring shows the production of exogenous lytic enzymes. The fourth ring illustrates antagonism against *Fusarium oxysporum* f.sp. *cubense* race 1 (CNRF-MIC17191) (AFO). Relative scores for PGP traits and lytic enzyme assays ranged numerically from zero to two. For the antagonism tests, inhibition percentage values obtained on the sixth day of confrontation were considered.

Finally, 38 strains (40%) exhibited antagonism against *F. oxysporum* (AFO) (Figure 3C) and as shown in Figure 4, these physiological activities are distributed across the different taxa.

Hence, the culturable rhizobacterial community associated with tunicate maize showed diverse and redundant physiological activities important for nutrition, ecology and health of the plant.

### Functional contributions of rhizobacteria at tasseling (VT) stage

Members of Oxalobacteraceae family displayed auxin production, P-solubilizers, putative diazotrophy, and antagonism; most prominent strains were *Herbaspirillum lusitanum* CPO E2-10 and CPO E3-28, and *Herbaspirillum seropedicae* CPO E3-1. Similarly, members of Comamonadaceae family demonstrated abilities for auxin biosynthesis, exogenous lytic enzyme production, and antagonism to phytopathogens; prominent strains were *Delftia acidovorans* CPO E1-16 and *Paracidovorax wauterssi* CPO E3-3.

All members of *Pseudomonadaceae* family excelled in auxin production, P-solubilizers, putative diazotrophy, siderophore production, and lytic enzyme synthesis (cellulase and protease); remarkable strains were *Pseudomonas fluorescens* CPO E2-1, CPO E3-27, and CPO E2-9, as well as strains *Pseudomonas oryzihabitans* CPO E1-11 and CPO E3-6. In the same way, all members of Rhizobiaceae family produced auxins and exogenous cellulase, and strains *Rhizobium grahamii* CPO E1-17 and *Rhizobium alamii* CPO E3-20 showed antagonism against *F*. *oxysporum* (Figure 3C), and strains *Rhizobium grahamii* CPO E3-24 and *Neorhizobium huautlense* CPO E1-7 were siderophore producers.

Several members of *Bacillaceae* family had capabilities as P-solubilizers and auxin producers along with lytic activities (cellulase and protease); the strain *Priestia megaterium* CPO 24A2 also displayed putative diazotrophy and biocontrol capabilities. Likewise, Micrococcaceae family revealed antagonistic and exogenous protease synthesis abilities; additionally, the strain *Pseudarthrobacter phenanthrenivorans* CPO E2-15 produced auxins and showed putative diazotrophy.

Finally, within the Xanthomonadaceae family, the strains *Stenotrophomonas indicatrix* CPO 12A2 and CPO 13A2 exhibited significant PGPR traits and lytic capabilities (Figure 4A).

Therefore, rhizobacteria from the culturable fraction at the VT stage exhibited multiple PGPR *in vitro* traits related to phytostimulation (IAA-producer), nutrient acquisition and competition (P-solubilizers, putative BNF, and siderophore production), as well as protection against phytopathogens (AFO, siderophore production, and lytic enzymes) (Figure 4A).

### Functional contributions of rhizobacteria at maturity/senescence (R6) stage

Actinobacteria belonging to the Microbacteriaceae and Micrococcaceae families, predominant in the rhizosphere isolates of mature/senescent plants, exhibited remarkable lytic activities (Figure 4B). Notably, the strains *Microbacterium natoriense* CPO E4-8, *M. suwonense* CPO 15A3 and CPO 16A3, *M. hominis* CPO E4-15, *M.* sp. CPO 40-2A3, *Leifsonia xyli* CPO 40A3 and CPO 13A3, *Agromyces atrinae* CPO E4-22, *Arthrobacter oryzae* CPO 12A3, *Arthrobacter senegalensis* CPO 5A3, and *Arthrobacter* sp. CPO 52A3 and CPO 1A3 demonstrated at least two of the analyzed proteolytic, lipolytic, or cellulolytic activities. Particularly, *Microbacterium* strains (family Microbacteriaceae) were found almost exclusively at this phenological stage. Within this collection of actinobacteria, the strains *Microbacterium natoriense* CPO E4-8 and *M. hominis* CPO E4-15 also exhibited important PGPR traits.

Likewise, *Arthrobacter* strains (family Micrococcaceae) were detected in both the VT and R6 stages. Despite exhibiting synthesis of lytic enzymes, mainly proteases, the *Arthrobacter* strains isolated at the R6 stage did not show inhibition against *F. oxysporum*, whereas such inhibition was observed in those isolates from the VT stage (Figure 4).

Finally, we also found Proteobacteria with notable PGPR capabilities and antagonism against pathogens. Most prominent isolates were *Caballeronia arvi* CPO E4-7, *Stenotrophomonas* sp. CPO E4-29, *Sphingobium fuliginis* CPO E5-1 and CPO E5-15, *Ochrobactrum intermedium* CPO E5-5 and CPO E5-24, *Herbaspirillum seropedicae* CPO E5-14, *Pseudomonas brassicacearum* CPO E5-18, and *Paraburkholderia caledonica* CPO E6-4 (Figure 4B).

Overall, rhizobacteria obtained from the culturable fraction at the R6 stage showed important lytic activities and notable PGPR traits.

## Discussion

The soil of Ixtenco *milpas* consists of sandy-loam with slightly acidic pH, a moderate percentage of SOM content, and exhibits deficiencies in nitrogen (N), potassium (K), and phosphorus (P) in some plots. Additionally, the soil has a low cation exchange capacity (CEC), indicating a low nutrient reserve. Consequently, tunicate maize, like other maize landraces (Higdon et al., 2020; Guzzon et al., 2021) typically grow under suboptimal soil conditions in spite of the agricultural practices implemented in the Ixtenco *milpas* (Huato et al., 2013; Orozco-Bolaños et al., 2019). This suggests that organic compound recycling is still at an early stage, requiring the optimization of agroecosystems through the management of microbial agrobiodiversity to enhance carbon and nutrient cycles, thereby improving the availability and retention of N and P and impacting soil quality (Mosier et al., 2021).

The assembly patterns of the rhizosphere microbiome are influenced by abiotic factors (e.g., soil properties, agricultural practices and climate) and plant-specific biotic factors (e.g., nutritional status and requirements, physical and phenological stages) (Xiong et al., 2021; Xun et al., 2021). Predominant phyla of rhizosphere bacterial community isolated from tunicate maize were Proteobacteria and Actinobacteria, followed by Firmicutes and Bacteroidetes. This distribution aligns with findings from various metagenomic studies, which reveal the dominance of these phyla in the relative abundance within rhizosphere bacterial communities associated to maize (Peiffer et al., 2013; Walters et al., 2018; Brisson et al., 2019; Huang et al., 2022; Singh and Goodwin, 2022).

Since the rhizosphere soil samples originated from the same edaphoclimatic region, with similar agronomical practices, and both samples generally exhibited similarities in physical and chemical characteristics, the differences in the enrichment proportions of Proteobacteria and Actinobacteria phyla observed between bacteria isolated from Ixtenco I and Ixtenco II locations, could be attributed to variations in nutritional needs of plants at the studied phenological stages (VT and R6, respectively), as well as to eco-physiological functions of the recorded rhizobacteria. In this regard, the release of low molecular weight carbon compounds (easily degradable) from roots has been linked to the stimulation of a dominant bacterial community in the rhizosphere (primarily composed of copiotrophs belonging to Proteobacteria and Bacteroidetes) throughout plant growth (Ling et al., 2022). Conversely, the oligotrophic community tends to dominate the rhizosphere environment at post-harvest stages when only recalcitrant and complex carbon compounds (cellulose, hemicelluloses, lignin) are dominant (Zheng et al., 2021). Actinobacteria are associated to the degradation of plant residues and the recycling of SOM during later stages of wheat straw decomposition (Bastian et al., 2009), particularly due to their lytic capabilities of lignocellulose residues (Boukhatem et al., 2022).

The rhizosphere bacterial community isolated from tunicate maize grown in agroecosystem at the VT stage was dominated by the families Oxalobacteraceae, Micrococcaceae, Comamonadaceae, Bacillaceae, Pseudomonadaceae, and Rhizobiaceae. These exhibited *in vitro* physiological activities related to phytohormone stimulation, nutrient acquisition, and competition, and protection against phytopathogens. The prevalence of these taxa is attributed to that recruitment mediated by both plant rhizodepositions and agricultural practices implemented at Ixtenco agroecosystems. Supporting this, a metagenomic study of maize rhizobacterial communities at different growth stages, cultivated in soil under conventional and conservation agricultural practices, reported the enrichment of fast-growing copiotrophic bacteria belonging to Rhizobiales, Sphingomonadales, Xanthomonadales, and Burkholderiales, with rhizospheric functions primarily related to degradation, utilization, assimilation, and biosynthesis. This taxonomic and functional diversity was attributed primarily to conservation agricultural practices following the rhizosphere effect during specific phenological stages, such as flowering and grain filling (Navarro-Noya et al., 2022).

The rhizosphere soil from which bacteria were isolated showed a marked deficiency in TN and during tasseling stage, plants typically exhibit a high demand for N (Rhezali and Lahlali, 2017). About this, a previous study under N-limiting conditions observed that flavones derived from roots of certain maize varieties promoted the rhizosphere enrichment of the Oxalobacteraceae family to stimulate nutrient uptake and growth (Yu et al., 2021). While some members of Rhizobiaceae family are well-known symbiotic bacteria in legumes (Ramirez-Puebla et al., 2019), the species isolated in this study (*Rhizobium grahamii, Rhizobium alamii, Neorhizobium huautlense* and *Rhizobium dioscoreae*) have not been reported previously as symbionts of Ayocote bean (*Phaseolus coccineus*), a legume used in the Ixtenco *milpas*, or as rhizospheric associates of maize landraces. This study, therefore, represents the first report of these species associated with rhizosphere of native maize. However, species such as *Rhizobium grahamii* can nodulate various legumes (Peralta et al., 2016) and other rhizobia are common endophytes in certain maize cultivars, where they positively influence nitrogen acquisition and promote plant growth (Rosenblueth et al., 2018; Ramirez-Puebla et al., 2019).

Moreover, members of the Comamonadaceae and Pseudomonadaceae families present at VT stage may play roles in both growth promotion and the mitigation of abiotic stress in tunicate maize. This is supported by reports in which these two families are enriched in maize roots in response to chilling stress, exerting positive effects on the development of young maize plants under low-temperature conditions (Beirinckx et al., 2020). Additionally, a study on maize landraces from arid regions found that the *Pseudomonas* genus was the most abundant member of those culturable seed-endophytic bacterial communities, which are inducing drought tolerance during early developmental stages (Arellano-Wattenbarger et al., 2024).

Similarly, a comparative metagenomic study identified the Bacillaceae family as dominant in the rhizosphere of teosinte, landraces, and inbred lines of maize during flowering and maturity/senescence stages, but not at seedling stage (Huang et al., 2022).

On the other hand, at the R6 stage, when the plant has completed its life cycle and aerial plant residues fall to the soil surface, labile carbon compounds in the rhizosphere significantly decrease and are succeeded by recalcitrant carbon compounds in the rhizosphere environment (Zheng et al., 2021). In this context, where the rhizospheric effect is no longer decisive in microbial recruitment, the culturable rhizobacterial community exhibited a pronounced enrichment of the Microbacteriaceae family, particularly the genus *Microbacterium*, which displays profiles of lytic enzyme production targeting polymeric biomolecules. This suggests a potential ecological function in the degradation of complex carbon compounds, a critical initial step in the mineralization of organic matter and nutrient recycling. This aspect is further supported by findings by Chiba et al. (2021), who highlighted the correlation between the rates of litter decomposition during its early phases and the abundance of bacteria classified as Sphingomonadaceae, Microbacteriaceae, and Burkholderiaceae.

Additionally, the Micrococcaceae family (mainly the genus *Arthrobacter*) was registered at both VT and R6 stages and exhibited capabilities of producing auxins and lytic enzymes; therefore, the members of this family could play physiological roles at plant nutrition and ecological levels like nutrient cycling in soil. This suggestion is supported by: (i) bacterial descriptions from the genus *Arthrobacter* as plant growth promoters under stressful conditions and as degraders of polymeric compounds including xenobiotic compounds (Roy and Kumar, 2020); and (ii) information provided by Fu et al. (2022) regarding the versatile copiotrophic-oligotrophic behavior of some *Arthrobacter* species.

Interestingly, the antibiosis observed in certain *Arthrobacter* strains at the VT stage, but not in those recorded at R6 stage, may not be related to their lytic activities but rather to the production of one or more bioactive compounds effective against phytopathogens. For instance, *Arthrobacter phenanthrenivorans* Se32.02 has been reported to inhibit the growth of *Rhizoctonia solani* through the secretion of antifungal molecules (Santos et al., 2020). Additionally, *A. kerguelensis* VL-RK_09 inhibits the growth of fungi, bacteria, and yeasts by secreting the bioactive compound S,S-dipropyl carbondithioate (Munaganti et al., 2016). Recently, antifungal compounds such as arthropeptide A and B, derived from *A. psychrophenolicus* and *A. humicola*, respectively, have also been identified (Ramlawi et al., 2021; Gomez et al., 2023). Therefore, these strains could represent a potential source of bioactive compounds for biological control.

Likewise, the community of Proteobacteria recorded at R6 stage (with capabilities for producing auxin and lytic enzymes and exerting fungal pathogen suppression) could also play ecological roles in nutrient cycling and control of fungal pathogens. Although some Proteobacteria genera are associated with cellulolytic activity for degrading corn residues during the silage process (Ramírez-Vega et al., 2020), the present is study represents the first report about potential PGPR isolated at the maturity/senescence phenological stage (dry weight material) from tunicate maize landrace, including *Sphingobium fuliginis*, *Ochrobactrum intermedium*, *Herbaspirillum seropedicae*, *Pseudomonas brassicacearum*, and *Paraburkholderia caledonica*.

Notably, 57% of rhizobacteria produced IAA, a key phytohormone that regulates plant development, stimulates root formation, enhances exudation, and increases the availability of soil microbes interacting with roots (Backer et al., 2018). Several IAA-producing genera identified in tunicate maize, such as *Arthrobacter* (Angulo et al., 2014), *Bacillus*, *Herbaspirillum* (Yan et al., 2018), *Ochrobactrum* (Saini et al., 2017), *Pseudomonas* (Cantabella et al., 2021), *Rhizobium* (Zahir et al., 2010; Zhao et al., 2018), and *Stenotrophomonas* (Kumar et al., 2023), have previously been noted for their auxin biosynthetic capabilities.

Phosphate solubilization (PS), a PGPR trait exhibited by certain microorganisms, enables the conversion of insoluble soil phosphate into bioavailable soluble phosphate for plants (Gouda et al., 2018). The PS activity was observed in 26% of isolates, with *Pseudomonas*, followed by *Herbaspirillum*, *Priestia*, and *Paraburkholderia* strains exhibiting this trait. These findings align with previous reports, which indicate that while various bacterial genera possess phosphate-solubilizing capacities, strains of *Pseudomonas* are particularly efficient (Villegas and Fortin, 2001; Li et al., 2017a; Saeid et al., 2018). Similarly, biological nitrogen fixation (BNF) is a highly sought-after PGPR trait for developing bioinputs targeting cereals, as nitrogen is a critical and limiting nutrient for their growth, development, and productivity (Rosenblueth et al., 2018). In the present study, 19% of the isolates exhibited putative diazotrophy. Some species among the identified *Herbaspirillum*, *Pseudomonas*, *Arthrobacter*, *Novosphingobium*, and *Microbacterium* strains have been reported as nitrogen-fixing (Rangjaroen et al., 2017; Rilling et al., 2018; Boukhatem et al., 2022). Specifically, *Herbaspirillum seropedicae*, *H. lusitanum*, and *Pseudomonas oryzihabitans* have been recognized as efficient free-living diazotrophs in rice, maize, sorghum, sugarcane, and bananas crops (Baldani et al., 1992; Valverde et al., 2003; Ngamau et al., 2012; Rosenblueth et al., 2018).

Siderophores are iron-chelating compounds synthesized by microbes that play a crucial role in plant nutrition, microbial competition, and disease suppression. By sequestering iron, bacterial siderophores limit the availability of this essential element to phytopathogens, thereby inhibiting their growth and colonization of plant (Timofeeva et al., 2022). In the present study, 25% of the bacterial strains produced siderophores, with these strains belonging primarily to various genera, including *Pseudomonas, Stenotrophomonas, Paraburkholderia, Sphingobium, Ochrobactrum, Rhizobium,* and *Neorhizobium*. *Pseudomonas* species, commonly found in the rhizosphere, are reported to produce various classes of siderophores which include pyoverdines, pseudobactin, and pyochelins (Crowley, 2006), moreover, *Stenotrophomonas*, *Paraburkholderia*, and *Rhizobium* strains produced enterobactin (Hisatomi et al., 2021), gramibactin (Hermenau et al., 2018) and catechol siderophores (Datta and Chakrabartty, 2014), respectively.

Hydrolytic enzymes are widely distributed among the isolated taxa and may play relevant role in organic matter decomposition and nutrient mineralization in organically managed soils (Gao et al., 2024), such as those in Ixtenco, as well as in the suppression of diseases and damage caused by phytopathogenic fungi and insects (Riseh et al., 2024). In the present investigation, 65% of the bacterial strains produced protease, 55% produced cellulase, and 22% produced lipase, while none produced chitinase. *Pseudomonas*, *Arthrobacter*, *Paracidovorax*, *Stenotrophomonas*, *Microbacterium,* and *Priestia* strains stood out for their multiple lytic activities and have previously been noted for their production of cell-wall degrading enzymes related to antagonism (Pathma and Sakthivel, 2013; Fira et al., 2018; Bonaterra et al., 2022; Ebrahimi-Zarandi et al., 2022). In this context, 38 strains exhibited antagonism against *F. oxysporum* (AFO), with 79% presenting at least one lytic activity and 34% producing siderophores (Figure 3C), suggesting the importance of these mechanisms in the inhibition of phytopathogens (Riseh et al., 2024). Other suppression mechanisms not explored in this study such as the production of HCN and bioactive antifungal compounds could be involved (Choudhary et al., 2022).

In our study, the culturable rhizobacterial community associated with tunicate maize, comprised 42 genera, and exhibited diverse and redundant physiological activities related to phytostimulation, nutrient acquisition, pathogen protection, and nutrient cycling. This functional diversity may be attributed to agricultural practices utilized for cultivating tunicate maize. In this regard, it has been demonstrated that organically fertilized agroecosystems are particularly effective in sustaining soil health (Yang et al., 2024) by fostering microbial diversity and interactions, broadening microbial metabolic capacities, enhancing functional redundancy, suppressing phytopathogenic microbes, and facilitating the mineralization of organic compounds within the maize rhizosphere (Luo et al., 2018; Kebede, 2021; Gao et al., 2024).

Specifically, strains identified in this study as inoculant candidates included *Priestia megaterium* which has been utilized as bio-inoculant in maize (Moturu et al., 2023); *Herbaspirillum seropedicae* and *H. lusitanum* are recognized as efficient free-living diazotrophic bacteria in Poaceae plants such as maize, rice (Baldani et al., 2000), and sugarcane (Oliveira et al., 2009); *Ochrobactrum intermedium* has been reported to enhance the productivity of rice under salinity stress (Sultana et al., 2020), produce IAA and siderophores, and exhibit ACC deaminase activity in peanut under abiotic stresses (Paulucci et al., 2015), as well as display biopesticide activity in sugarcane (Hassan et al., 2014); *Rhizobium grahamii* is a newly discovered rhizobia species endemic to Mexico, capable of symbiotically fixing nitrogen in various legumes including *Leucaena leucocephala* and *Phaseolus vulgaris* (Althabegoiti et al., 2014; Ramirez-Puebla et al., 2019); *Rhizobium alamii* has been described as an exopolysaccharide-producing rhizobia that enhances drought tolerance in non-leguminous species (e.g., rapeseed and sunflower) (Alami et al., 2000; Tulumello et al., 2021); *Neorhizobium huautlense* has been shown to improve the yield of rice and hot pepper while also functioning in the bioremediation of heavy metal-contaminated soil (Chen et al., 2016; Li et al., 2017b).

Furthermore, *Pseudomonas fluorescens*, *P. oryzihabitans,* and *P. brassicacearum* may act as biocontrol agents of phytopathogens and plant growth promoters (Horuz, 2021; Raio, 2024); however, in this study, only *P. brassicacearum* showed significant antagonistic capability against *F. oxysporum,* potentially through the secretion of secondary bioactive metabolites (e.g., hydrogen cyanide and a complex mixture of phloroglucinol derivates) (Nelkner et al., 2019; Biessy and Filion, 2021). *Delftia acidovorans* is a PGPR utilized as part of inocula for canola and soybean (Suchan et al., 2020). *Stenotrophomonas indicatrix* has been isolated and evaluated in sunflower plants as promising PGPR due to its abilities as PS, and as producer of siderophores, auxins and multiple hydrolytic enzymes (Adeleke et al., 2021). *Microbacterium natoriense* has been described as diazotrophic from phylloplane of wheat varieties (Batool et al., 2016).

Finally, *Sphingobium fuliginis* and *Pseudarthrobacter phenanthrenivorans* are understudied in agricultural sciences and these species have not been previously described as PGPR; however, they are reported as degraders of several recalcitrant aromatic compounds such as alkylphenols, biphenols (Kuroda et al., 2017), phenol (Asimakoula et al., 2023), 4-hydroxybenzoic acid (Tsagogiannis et al., 2024), and phenanthrene (Li et al., 2024), thus, highlighting a perspective for future research for biotechnological applications.

Consequently, this bioprospection effort focused on identifying PGP traits under *in vitro* conditions within the culturable rhizobacterial community associated with tunicate maize during tasseling and maturity/senescence plant stages. This experimental approach facilitated the detection and acquisition of a valuable functional bacterial germplasm in an unexplored agroecosystem, which holds significant potential for the development of future bioinoculants aimed at enhancing the sustainable productivity and economic viability of milpa systems in the central Mexican highlands.

## Conclusion

This research marks the first exploration of culturable bacteria associated with the rhizosphere of tunicate maize landrace grown in an agroecosystem, representing a unique ecological niche shaped by the agricultural practices and soil nutrient deficiencies under which it is cultivated.

Tunicate maize harbors functional groups of culturable indigenous rhizobacteria with physiological activities related to plant nutrition, health, and ecology. The nutritional requirements associated with the phenology of plant and agricultural practices implemented, influence the diversity and redundance functionality of culturable rhizosphere populations.

The culturable population during tasseling stage exhibited capabilities for stimulating plant growth and nutrition, as well as inhibiting fungal phytopathogens, while the culturable population in maturity/senescence stage showed lytic activities relevant to the mineralization of organic matter and nutrient recycling. Additionally, we detected possible novel rhizospheric functional bacterial species such as *Rhizobium*, *Sphingobium*, and *Arthrobacter* which are not previously described in maize landraces, as well as the bioprospection of potential PGPR detected at plant phenological stages poorly explored (like maturity/senescence).

Several indigenous bacterial strains exhibited multiple plant growth-promoting activities, suggesting their potential as biofertilizers and/or biocontrol agents to promote sustainable agriculture. However, further complementary studies are needed to assess their biological effectiveness in plants. Furthermore, this study contributes to the development of a knowledge framework that may provide the importance of the conservation of endangered maize landraces through the recognition and promotion of their associated microbial genetic resources. This culturable approach facilitates the development of customized bioformulations tailored to local agroecosystems, particularly for application in the sustainable intensification of production systems in the central Mexican highlands.

## Data Availability

The datasets presented in this study can be found in online repositories. The names of the repository/repositories and accession number(s) can be found in the article/Supplementary material.
