# Supplementary material for: Evaluation of functional plant growth-promoting activities of culturable rhizobacteria associated to tunicate maize (Zea mays var. tunicata A. St. Hil), a Mexican exotic landrace grown in traditional agroecosystems
Source: Front Microbiol. 2024 Oct 2;15:1478807. doi: 10.3389/fmicb.2024.1478807 (PMC11480017; doi:10.3389/fmicb.2024.1478807)
Supplement: Supplementary file 1 [file Table_1.docx]

**SUPPLEMENTAL MATERIAL**

**TABLE S1.**

Morphological characterization and molecular identification of rhizospheric bacterial strains isolated from tunicate maize.

|  | **16s rRNA molecular identification** | | | | **Morphology** | | **Isolation** |
| --- | --- | --- | --- | --- | --- | --- | --- |
| **Strain** | **GenBank accession number** | **Identified microorganism** | **Identity percentage (%)** | **Closest related accession number in NCBI database** | **GS** | **Colony morphology** | **phenological stage/culture medium** |
| CPO E1-2  CPO E1-4  CPO E1-6  CPO E1-7  CPO E1-9  CPO E1-10  CPO E1-11  CPO E1-16  CPO E1-17 | PP111633  PP111634  PP111635  PP111636  PP111637  PP111638  PP111639  PP111640  PP111641 | *Mucilaginibacter rubeus*  *Paracidovorax wautersii*  *Arthrobacter globiformis*  *Neorhizobium huautlense*  *Dyadobacter fermentans*  *Mucilaginibacter rubeus*  *Pseudomonas oryzihabitans*  *Delftia acidovorans*  *Rhizobium grahamii* | 98.59  99.52  99.79  99.56  97.14  100  99.86  100  99.92 | MK131346 MF062564  KY649415  OQ195987  LN890052  NR_157717  AB675634  AM180725  NR_118140 | G-  G-  G+  G-  G-  G-  G-  G-  G- | W, Tr, M, R, C, Sm  W, Op, S, R, C, Sm  W, Op, M, R, C, G, Sm  W, Tr, S, R, C, Sm  Y, T, M, R, C, Sm  P, Tr, S, R, C, Sm  Y, Tr, L, I, F, Sm  Y, Tr, M, I, C, Sm  W, Op, L, I, C, G, Sm | Tasseling/YMA  Tasseling/YMA  Tasseling/YMA  Tasseling/YMA  Tasseling/YMA  Tasseling/YMA  Tasseling/YMA  Tasseling/YMA  Tasseling/YMA |
| CPO E2-1  CPO E2-2  CPO E2-3  CPO E2-4  CPO E2-5  CPO E2-7  CPO E2-9  CPO E2-10  CPO E2-12  CPO E2-15  CPO E2-16  CPO E2-20 | PP111642  PP111643  PP111644  PP111645  PP111646  PP111647  PP111648  PP111649  PP111650  PP111651  PP111652  PP111653 | *Pseudomonas fluorescens*  *Herbaspirillum lusitanum*  *Xanthomonas* sp.  *Microbacterium paraoxydans*  *Herbaspirillum hiltneri*  *Roseateles* sp.  *Pseudomonas fluorescens*  *Herbaspirillum lusitanum*  *Pseudacidovorax intermedius*  *Pseudarthrobacter phenanthrenivorans*  *Herbaspirillum lusitanum*  *Herbaspirillum lusitanum* | 100  99.73  99.32  100  99.93  97.70  100  99.72  100  100  99.73  99.73 | DQ453823  NR_028859  JX997975  MH669308  KU305712  NR_157656  DQ453823  NR_028859  NR_044241  LN890039  NR_028859  NR_028859 | G-  G-  G-  G+  G-  G-  G-  G-  G-  G+  G-  G- | Cr, Op, L, R, C, Sm  T, M, R, C, Sm  Y, Tr, S, R, C, Sm  Y, Tr, M, I, C, Sm  W, Tr, M, R, C, Sm  W, Op, L, R, C, G, Sm  W, Op, M, I, C, G  Cr, Tr, M, R, C, Sm  O, Op, M, R, Ra, Sm  Cr, Op, M, R, C, G, Sm  T, M, R, C, Sm  T, M, R, C, Sm | Tasseling/YMA  Tasseling/YMA  Tasseling/YMA  Tasseling/YMA  Tasseling/YMA  Tasseling/YMA  Tasseling/YMA  Tasseling/YMA  Tasseling/YMA  Tasseling/YMA  Tasseling/YMA  Tasseling/YMA |
| CPO E3-1  CPO E3-2  CPO E3-3  CPO E3-5  CPO E3-6  CPO E3-9  CPO E3-13  CPO E3-17  CPO E3-20  CPO E3-21  CPO E3-22  CPO E3-24  CPO E3-26  CPO E3-27  CPO E3-28 | PP111654  PP111655  PP111656  PP111657  PP111658  PP111659  PP111660  PP111661  PP111662  PP111663  PP111664  PP111665  PP111666  PP111667  PP111668 | *Herbaspirillum seropedicae*  *Novosphingobium* sp.  *Paracidovorax wautersii*  *Flavobacterium sp.*  *Pseudomonas oryzihabitans*  *Pseudarthrobacter chlorophenolicus*  *Neobacillus drentensis*  *Arthrobacter* sp.  *Rhizobium alamii*  *Sphingobium* sp.  *Paracidovorax wautersii*  *Rhizobium grahamii*  *Rugamonas aquatica*  *Pseudomonas fluorescens*  *Herbaspirillum lusitanum* | 99.58  100  99.46  98.60  99.86  99.86  99.93  99.85  99.86  99.93  99.45  99.92  99.31  99.77  99.73 | LC191533  JN613484  MF062564  AM922192  AB675634  NR_074518  JF496444  MN865732  LC106996  AY689029  MF062564  NR_118140  MW164970  DQ453823  NR_028859 | G-  G-  G-  G-  G-  G+  G+  G+  G-  G-  G-  G-  G-  G-  G- | W, Op, M, R, C, G, Sm  Y, Tr, S, R, C, Sm  W, Tr, M, R, C, Sm  Y, Tr, L, I, C, Sm  Y, Op, M, R, F, Ro, D  W, Op, L, R, C, G, Sm  W, Op, M, I, Ra, Ro, D  W, Tr, M, R, C, G, Sm  T, L, I, C, Sl, Mu  Y, Tr, S, R, C, Sm  W, Tr, M, R, C, Sm  W, Op, L, R, C, G, Sm  Pu, Tr, M, R, F, D  Cr, Tr, M, R, Ra, Sm  T, M, R, C, Sm | Tasseling/YMA  Tasseling/YMA  Tasseling/YMA  Tasseling/YMA  Tasseling/YMA  Tasseling/YMA  Tasseling/YMA  Tasseling/YMA  Tasseling/YMA  Tasseling/YMA  Tasseling/YMA  Tasseling/YMA  Tasseling/YMA  Tasseling/YMA  Tasseling/YMA |
| CPO 1A2  CPO 2A2  CPO_4A2  CPO 5_3A2  CPO_7A2  CPO_8A2  CPO 9A2  CPO_12A2  CPO 13A2  CPO_15A2  CPO 16A2  CPO_22A2  CPO_23A2  CPO 24A2  CPO_27A2  CPO 30A2  CPO_34A2 | PP111669  PP111670  PP111671  PP111672  PP111673  PP111674  PP111675  PP111676  PP111677  PP111678  PP111679  PP111680  PP111681  PP111682  PP111683  PP111684  PP111685 | *Micrococcus yunnanensis*  *Arthrobacter globiformis*  *Priestia megaterium*  *Paenibacillus zeae*  *Paenibacillus zeae*  *Neobacillus niacini*  *Streptomyces chattanoogensis*  *Stenotrophomonas indicatrix*  *Stenotrophomonas indicatrix*  *Peribacillus frigoritolerans*  *Chitinophaga arvensicola*  *Priestia megaterium*  *Staphylococcus equorum*  *Priestia megaterium*  *Arthrobacter globiformis*  *Alcaligenes faecalis*  *Acinetobacter johnsonii* | 99.86  99.73  99.74  98.61  99.14  99.44  100  100  99.87  99.80  99.03  99.80  99.93  100  99.67  99.61  99.74 | KT443901  AB098573  OQ559533  KP965583  KP965583  OM971476  FJ171335  CP079106  CP079106  CP128118  AM237312  OQ559533  CP013980  OQ559533  AB098573  CP031012  CP010350 | G+  G+  G+  G+  G+  G+  G+  G-  G-  G+  G-  G+  G+  G+  G+  G-  G- | Cr, Op, S, R, C, Sm  W, Op, M, R, C, G, Sm  W, Op, L, I, F, Sm  W, Op, L, I, F, D  W, Op, L, I, Ra, D  Cr, Tr, M, Sm  W, Op, M, R, F, Wr, D  W, Tr, M, R, C, Sm  W, Tr, S, R, C, Mu  Cr, Op, S, R, C, Sm  O, Tr, M, R, C, Sm  W, Op, M, I, F, Sm  W, Tr, L, I, F, Sm  Cr, Op, M, I, F, Sm  W, Op, S, R, C, G, Sm  W, Op, S, R, C, Sm  W, Op, L, I, F, Sm | Tasseling/NA  Tasseling/NA  Tasseling/NA  Tasseling/NA  Tasseling/NA  Tasseling/NA  Tasseling/NA  Tasseling/NA  Tasseling/NA  Tasseling/NA  Tasseling/NA  Tasseling/NA  Tasseling/NA  Tasseling/NA  Tasseling/NA  Tasseling/NA  Tasseling/NA |
| CPO E4-1  CPO E4-2  CPO E4-4  CPO E4-7  CPO E4-8  CPO E4-13  CPO E4-15  CPO E4-22  CPO E4-29  CPO E4-32 | PP111686  PP111687  PP111688  PP111689  PP111690  PP111691  PP111692  PP111693  PP111694  PP111695 | *Chryseobacterium indoltheticum*  *Microbacterium oxydans*  *Stenotrophomonas maltophilia*  *Caballeronia arvi*  *Microbacterium natoriense*  *Sphingobacterium siyangense*  *Microbacterium hominis*  *Agromyces atrinae*  *Stenotrophomonas* sp.  *Rhizobium dioscoreae* | 99.65  100  99.85  99.38  100  99.52  99.89  100  100  98.64 | NR_042926  MT533951  MN826555  NR_145594  LC040930  EU373423  MH180761  NR_116743  LC133723  NR_179313 | G-  G+  G-  G-  G+  G-  G+  G+  G-  G- | O, Tr, S, R, C, Sm  Y, Tr, M, R, F, Sl  Y, Tr, M, R, C, Sm  Cr, Op, L, R, C, G, Sm  O, Tr, L, R, C, Mu  Cr, Tr, M, R, C, Sm  W, Tr, L, R, C, Mu  Y, Tr, L, I, C, Mu  T, S, R, C, Sm  W, Tr, L, R, C, Sl | Maturity/Senescence/YMA  Maturity/Senescence/YMA  Maturity/Senescence/YMA  Maturity/Senescence/YMA  Maturity/Senescence/YMA  Maturity/Senescence/YMA  Maturity/Senescence/YMA  Maturity/Senescence/YMA  Maturity/Senescence/YMA  Maturity/Senescence/YMA |
| CPO E5-1  CPO E5-5  CPO E5-6  CPO E5-9  CPO E5-11  CPO E5-13  CPO E5-14  CPO E5-15  CPO E5-18  CPO E5-21  CPO E5-23  CPO E5-24 | PP111696  PP111697  PP111698  PP111699  PP111700  PP111701  PP111702  PP111703  PP111704  PP111705  PP111706  PP111707 | *Sphingobium fuliginis*  *Ochrobactrum intermedium*  *Microbacterium esteraromaticum*  *Chryseobacterium daeguense*  *Brevundimonas* sp.  *Brevundimonas* sp.  *Herbaspirillum seropedicae*  *Sphingobium fuliginis*  *Pseudomonas brassicacearum*  *Pedobacter panaciterrae*  *Paraburkholderia graminis*  *Ochrobactrum intermedium* | 99.11  100  100  100  99.71  99.86  99.58  99.04  99.93  99.86  99.93  100 | OL423547  MT373519  MN685266  NR_044069  MF405110  MF405110  LC191533  OL423547  AJ292381  NR_041371  HQ698910  MK344317 | G-  G-  G+  G-  G-  G-  G-  G-  G-  G-  G-  G- | Y, Tr, S, R, C, Sm  W, Tr, L, I, C, Sm  T, S, R, F, D  Y, Tr, M, I, F, Sl  T, S, R, C, Sm  T, M, R, C, Sm  T, M, R, C, Mu  Y, Tr, M, R, C, G, Sm  Cr, M, R, C, Sm  Cr, M, R, C, Sm  W, Tr, L, I, C, G, Sl  W, Tr, M, I, F, G, Sm | Maturity/Senescence/YMA  Maturity/Senescence/YMA  Maturity/Senescence/YMA  Maturity/Senescence/YMA  Maturity/Senescence/YMA  Maturity/Senescence/YMA  Maturity/Senescence/YMA  Maturity/Senescence/YMA  Maturity/Senescence/YMA  Maturity/Senescence/YMA  Maturity/Senescence/YMA  Maturity/Senescence/YMA |
| CPO E6-4  CPO E6-13 | PP111708  PP111709 | *Paraburkholderia caledonica*  *Flavobacterium hydatis* | 100  95.74 | OP986441  LN613109 | G-  G- | W, Tr, L, G, Sl  Y, Tr, M, R, F, Sm | Maturity/Senescence/YMA  Maturity/Senescence/YMA |
| CPO_1A3  CPO_3A3  CPO_5A3  CPO_12A3  CPO_13A3  CPO_15A3  CPO_16A3  CPO_22A3  CPO_24A3  CPO_25A3  CPO_27A3  CPO_29A3  CPO_34_2A3  CPO_36A3  CPO_40-2A3  CPO_40A3  CPO_42A3  CPO_52A3 | PP111710  PP111711  PP111712  PP111713  PP111714  PP111715  PP111716  PP111717  PP111718  PP111719  PP111720  PP111721  PP111722  PP111723  PP111724  PP111725  PP111726  PP111727 | *Arthrobacter* sp.  *Paenibacillus xylanivorans*  *Arthrobacter senegalensis*  *Arthrobacter oryzae*  *Leifsonia xyli*  *Microbacterium suwonense*  *Microbacterium suwonense*  *Rhodanobacter fulvus*  *Leifsonia xyli*  *Microbacterium oxydans*  *Microbacterium foliorum*  *Microbacterium foliorum*  *Moraxella osloensis*  *Limnobaculum parvum*  *Microbacterium* sp.  *Leifsonia xyli*  *Sporosarcina* sp.  *Arthrobacter* sp. | 99.20  97.95  99.93  100  100  99.69  99.58  99.80  99.20  100  100  99.80  99.74  95.76  100  100  99.93  99.40 | JX164053  NR_178867  NR_179500  AB648956  GU332619  AP027728  AP027728  R_040952  CP014761  JX185498  CP041040  CP041040  CP024185.2  NR_176501  KX390640  GU332619  MN493889  CP113505 | G+  G+  G+  G+  G+  G+  G+  G-  G+  G+  G+  G+  G+  G-  G+  G+  G+  G+ | Cr, Op, M, R, C, Sm  W, M, R, C, Sm  W, M, I, F, Sm  Cr, M, I, F, Sm  Cr, M, R, C, G, Mu  W, M, R, C, Sm  W, S, R, C, Sm  Y, M, R, F, Sm  W, L, R, C, G, Mu  Y, S, R, F, Ro, D  Y, M, R, C, G, D  Cr, M, R, C, G, D  W, Tr, R, F, G, Sm  Cr, Tr, R, C, Sm  Y, Tr, R, C, G, D  Cr, L, R, C, Mu  Cr, Op, R, F, Sm  Y, Op, M, R, C, Sm | Maturity/Senescence/NA  Maturity/Senescence/NA  Maturity/Senescence/NA  Maturity/Senescence/NA  Mautrity/Senescence/NA  Maturity/Senescence/NA  Maturity/Senescence/NA  Maturity/Senescence/NA  Maturity/Senescence/NA  Maturity/Senescence/NA  Maturity/Senescence/NA  Maturity/Senescence/NA  Maturity/Senescence/NA  Maturity/Senescence/NA  Maturity/Senescence/NA  Maturity/Senescence/NA  Maturity/Senescence/NA  Maturity/Senescence/NA |

GS: gram staining test, positive (+) or negative (-). Colony morphology: color (Y: yellow, W: white, P: pink, O: orange, Cr: creamy-white, Pu: purple); opacity (T: Transparent, Tr: translucent, Op: opaque); size (L: large, M: medium, S: small); circular shape (R: regular, I: irregular); elevation (F: flat, C: convex, Ra: raised); surface (Sm: smooth, G: glistening, Ro: rough, Wr: wrinkled); texture (Sl: slimy, Mu: mucoid, D: dry). Culture medium where isolation and morphological characterization were performed: YMA (yeast mannitol agar plates) and AN (nutrient agar plates).
